# Exploring the cognitive and motor functions of the basal ganglia: an integrative review of computational cognitive neuroscience models

**DOI:** 10.3389/fncom.2013.00174

**Published:** 2013-12-06

**Authors:** Sebastien Helie, Srinivasa Chakravarthy, Ahmed A. Moustafa

**Affiliations:** ^1^Department of Psychological Sciences, Purdue UniversityWest Lafayette, IN, USA; ^2^Department of Biotechnology, Indian Institute of TechnologyMadras, India; ^3^Marcs Institute for Brain and Behaviour and School of Social Sciences and Psychology, University of Western SydneySydney, NSW, Australia

**Keywords:** basal ganglia, computational cognitive neuroscience, cognitive function, motor function, Parkinson’s disease

## Abstract

Many computational models of the basal ganglia (BG) have been proposed over the past twenty-five years. While computational neuroscience models have focused on closely matching the neurobiology of the BG, *computational cognitive neuroscience* (CCN) models have focused on how the BG can be used to implement cognitive and motor functions. This review article focuses on CCN models of the BG and how they use the neuroanatomy of the BG to account for cognitive and motor functions such as categorization, instrumental conditioning, probabilistic learning, working memory, sequence learning, automaticity, reaching, handwriting, and eye saccades. A total of 19 BG models accounting for one or more of these functions are reviewed and compared. The review concludes with a discussion of the limitations of existing CCN models of the BG and prescriptions for future modeling, including the need for computational models of the BG that can simultaneously account for cognitive and motor functions, and the need for a more complete specification of the role of the BG in behavioral functions.

## Introduction

The basal ganglia (BG) are a group of nuclei at the base of the forebrain that are strongly connected to the cortex. While the role of the BG had historically been restricted to motor function, a substantive amount of recent research suggests that the BG are also involved in a variety of cognitive functions (Steiner and Tseng, 2010). Behavioral and neural experiments with human and non-human animals have informed our understanding of the BG function for over a century, and the past two decades have seen an increased use of computational models to simulate the anatomy and functionality of the BG. The most anatomically detailed computational neuroscience models seldom go as far as simulating complex animal behavior (because of complexity issues), but simpler (less anatomically detailed) models can be used to simultaneously account for some anatomical details and complex animal behavior. The strength of these later *computational cognitive neuroscience* (CCN) models lies in that they can simultaneously account for both neuroscience data and behavioral data (Ashby and Helie, 2011).

This review article focuses on CCN models of the BG and classifies existing models according to cognitive and motor function. The remainder of this article is organized as follows. First, the anatomy that is usually included in CCN models of the BG is reviewed. This anatomy section is incomplete by design, as only details that are simulated to account for specific cognitive or motor function are included. Next, we review CCN models used to simulate six different cognitive functions, namely categorization, instrumental conditioning, probabilistic learning, working memory, sequence learning, and automaticity. This presentation is followed by CCN models of motor function. Computational cognitive neuroscience models of motor functions are separated into models of reaching, handwriting, and eye saccades. The review concludes with a discussion of the limitations of existing CCN models of the BG and prescriptions for future modeling. Future directions emphasize the need for CCN models of the BG that can simultaneously account for cognitive and motor functions, and the need for a more complete specification of the role of the BG in the reviewed functions.

## Neuroanatomy of the basal ganglia

**Figure 1:**
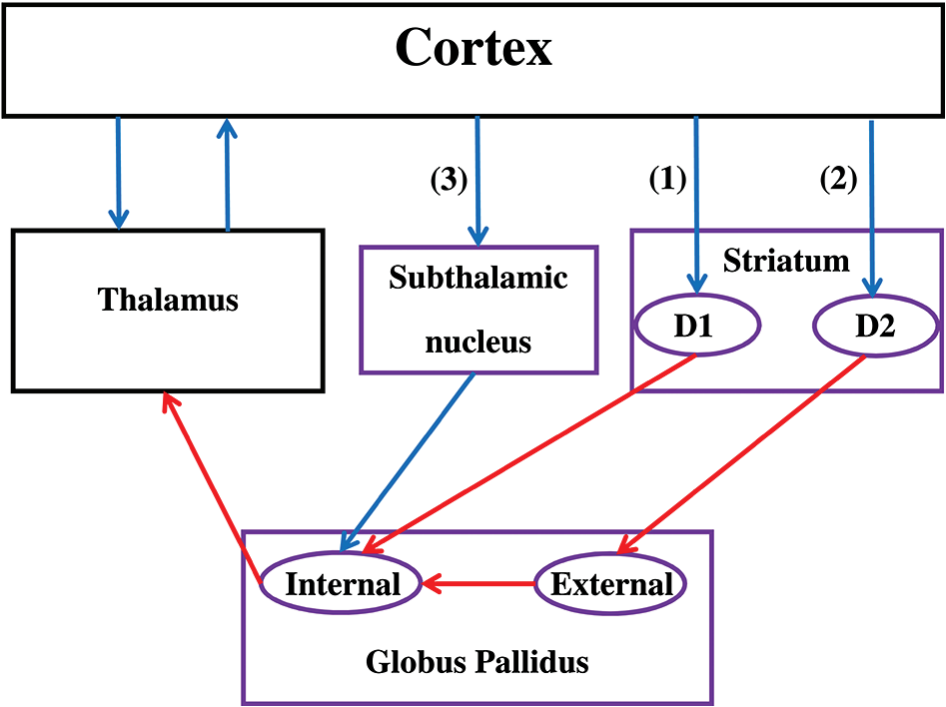
**Functional anatomy of the basal ganglia.** Note that only subdivisions included in most of the reviewed CCN models are represented. Purple boxes correspond to areas of the BG while black boxes are not included in the BG. Blue arrows represent excitatory (glutamatergic) connections while red arrows represent inhibitory (GABA) connections. The direct pathway **(1)** passes through the D1 receptors in the striatum, the indirect pathway **(2)** passes through the D2 receptors in the striatum, and the hyperdirect pathway **(3)** passes through the STN. If the thalamic projections target the same cortical region that initially targeted the striatum, the circuit is called a closed loop. Otherwise, the circuit is an opened loop.

The BG include the striatum (caudate, putamen, nucleus accumbens), the globus pallidus (GP), the subthalamic nucleus (STN), the substantia nigra (SN), the ventral tegmental area, and the olfactory tubercle (see Figure 1). The striatum receives the majority of afferent connections whereas the internal segment of the GP Globus pallidus (internal) (GPi) and SN pars reticulate (SNr) are the sources of the majority of efferent connections that target cortical regions via the thalamus. Based on both structural and functional evidence, the striatum is often divided into a ventral and a dorsal part. The ventral striatum includes the nucleus accumbens, ventromedial portions of the caudate and putamen, and the olfactory tubercle. The dorsal striatum, which is usually the main focus of CCN models of the BG, includes the remainder of the caudate and putamen.

Virtually all of the neocortex sends excitatory (glutamatergic) projections to the striatum (Reiner, 2010). Corticostriatal input is massively convergent with estimates ranging from 5,000 to 10,000 cortical neurons converging on a single striatal medium spiny neuron (MSN; the main striatal projection neurons) (Kincaid et al., 1998). Classically, corticostriatal organization is thought to follow a fairly strict spatial topography (Kemp and Powell, 1970). Along the rostral-to-caudal extent of the BG, the cortical afferents tend be more prevalent from rostral-to-caudal cortical regions. For instance, ventral striatum receives input predominantly from orbitofrontal cortex, ventromedial prefrontal cortex, and anterior cingulate cortex (ACC). As one moves caudally within the striatum, inputs from areas 9, 46, and 8 become more prevalent (Haber et al., 2006; Calzavara et al., 2007), followed by inputs from premotor regions (area 6) with the most caudal motor and somatosensory cortical regions projecting preferentially to the caudal putamen (Flaherty and Graybiel, 1994). Spatial topography holds as you continue rostrally and ventrally through parietal and temporal cortices as well as other extrastriate visual areas (Kemp and Powell, 1970; Yeterian and Pandya, 1993, 1995, 1998).

The thalamus provides another major source of input to the BG (Wilson, 2004), with the majority of thalamostriatal projections originating from the parafascicular-centromedian (CMPf) complex (Smith et al., 2010). Thalamic input to the striatum synapses on both MSNs and cholinergic tonically active neurons (TANs; a class of large-body interneurons) (Smith et al., 2004), with the latter likely playing an important role in modulating cortico-striatal synaptic plasticity (Ashby and Crossley, 2011). Finally, thalamic input to the striatum is in a position to modulate BG function by virtue of cortico-thalamo-striatal connections and striatal-thalamo-striatal feedback (Smith et al., 2010).

The BG also receives dopaminergic input that plays a critical role in modulating striatal activity. Dopamine is projected from the ventral tegmental area and SN pars compacta to the BG and prefrontal cortex, among other brain regions. Dopamine firing patterns fluctuate between two different modes: phasic and tonic. While the phasic mode is fast-acting and spans milliseconds, the tonic mode is long-acting and can span minutes or hours. The dissociable function of both phasic and tonic dopamine is debatable (Dreher and Burnod, 2002; Assadi et al., 2009; Moustafa et al., 2013). However, various studies suggest that phasic dopamine plays a key role in synaptic plasticity and reinforcement learning (Wickens et al., 1996; Reynolds et al., 2001), while tonic dopamine is important for speeding-up reaction times (Niv et al., 2007; Moustafa et al., 2008) and controlling the signal-to-noise ratio (Durstewitz and Seamans, 2008).

Information flow through the BG follows two distinct pathways (see Figure 1). Striatal MSNs in the direct pathway project directly to the output nuclei (e.g., GPi) and selectively express D1-like receptors (i.e., D1 and D5; Gerfen et al., 1990). The striatal MSNs in the indirect pathway project to the external segment of the GP Globus pallidus (external) (GPe) prior to reaching the output nuclei of the BG (e.g., GPi), and selectively express D2-like receptors (i.e., D2, D3, and D4; Gerfen and Young, 1988). In addition to the direct and indirect pathways, the STN is another major input structure of the BG receiving extensive cortical and thalamic input. This so-called hyperdirect pathway provides a means by which frontal cortical regions can monosynapically influence STN function (Nambu et al., 2002).

With abundant dopamine receptors in the BG affecting the dynamics of the different pathways, most CCN models of the BG include a role for dopamine. One important way of testing whether the hypothesized role for dopamine in the model is adequate is to simulate the model under dopamine-depleted conditions. Specifically, reducing the amount of dopamine available in the model should produce Parkinsonian symptoms. Parkinson’s disease (PD) is caused by the accelerated death of dopamine producing neurons. The pattern of cell loss is opposite to that of, and more severe than in, normal aging. Within the SN pars compacta, cell loss is predominately found in the ventral tier with less (but still extensive) damage in the dorsal tier (Fearnley and Lees, 1991; Gibb and Lees, 1991). In contrast, normal aging yields substantially less cell loss and in a dorsal-to-ventral pattern. Parkinsonian motor symptoms appear after a loss of 60–70% of SN pars compacta cells and 70–80% of dopamine levels in striatal nuclei (Bernheimer et al., 1973; Gibb and Lees, 1991). Motor symptoms include tremor, rigidity, bradykinesia, and akinesia. In addition to motor deficits, non-demented PD patients present cognitive symptoms that resemble those observed in patients with frontal damage. Numerous studies documenting cognitive deficits of PD patients have revealed impairment in a variety of tasks related to memory, learning, visuospatial skills, and attention (e.g., Gotham et al., 1988; Price et al., 2009).

## Cognitive function

While many cognitive functions have been attributed to the BG (for a review, see Steiner and Tseng, 2010), relatively few have been modeled and numerically simulated using CCN models, i.e., models that can simultaneously account for both neurobiological and behavioral data. Hence, this review does not constitute an attempt at reviewing all the cognitive and motor functions attributed to the BG: the focus is on CCN models of the BG. Note that the model descriptions included are conceptual, in that implementation details and mathematical formulations are not discussed. The reader is referred to the cited original papers for model details and equations. Table 1 summarizes the reviewed models along with their respective components.

**Table 1:** Summary of the basal ganglia components included in the reviewed models.

	DP (1)	IP (2)	HP (3)	Str	GPi	GPe	STN
**Cognitive**						
Ashby et al. (1998)	X			X	X		
Moustafa and Gluck (2011a)	X			X			
Ashby and Crossley (2011)	X			X	X		
Frank (2005)	X	X		X	X	X	
Guthrie et al. (2013)	X		X	X	X		X
Monchi et al. (2000)	X			X	X		
Ashby et al. (2005)	X			X	X		
Frank et al. (2001)	X			X	X		
Moustafa and Maida (2007)	X			X				
Schroll et al. (2012)	X		X	X	X	X	X
Nakahara et al. (2001)	X		X	X			
Ashby et al. (2007)	X			X	X		
Chersi et al. (2013)	X	X	X	X	*	X	X
**Motor**						
Bischoff (1998)	X	X		X	X	X	X
Magdoom et al. (2011)	X	X		X			
Gangadhar et al. (2008)		X				X	X
Contreras-Vidal and Stelmach (1995)	X	X	X	X	X	X	X
Dominey and Arbib (1992)	X			X	*		
Krishnan et al. (2011)	X	X		X	*	X	X

Note. DP = Direct pathway [(1) in Figure 1]; IP = Indirect pathway [(2) in Figure 1]; HP = Hyperdirect pathway [(3) in Figure 1]; Str = Striatum; GPi = Globus pallidus (internal); GPe = Globus pallidus (external); STN = Subthalamic nucleus. * These models used the substantia nigra pars reticulate (SNr) as their output node of the basal ganglia. In this context, the SNr is functionally equivalent to the GPi.

### Categorization

Categorization is the ubiquitous process by which individual items are grouped to form categories. The massive convergence of cortico-striatal connectivity makes the BG an ideal site for categorization, and much research supports the role of the BG in category learning (for a review, see Seger, 2008).

#### Models

One of the earliest and most prominent neurobiological models of categorization is called COVIS (Ashby et al., 1998). COVIS is a multiple-system theory that was originally developed to account for the many behavioral dissociations between verbal and non-verbal categorization (as described by the general recognition theory; Ashby and Gott, 1988). COVIS includes an hypothesis-testing system and a procedural learning system. The hypothesis-testing system can quickly learn a small set of (e.g., verbal) categories (those that can be found by hypothesis-testing and often be verbally described) while the procedural learning system can learn any type of arbitrary categories in a slow trial-and-error manner (e.g., non-verbal). Each categorization system relies on a separate brain circuit but, interestingly, they both include the BG. In the hypothesis-testing system, the BG is used to support working memory maintenance and for rule switching. In the procedural system, the BG is used to learn stimulus—response associations. The COVIS model of categorization has been used to simulate a large number of category learning experiments and made several behavioral predictions, many of which have been later confirmed by empirical experiments (for a review, Maddox and Ashby, 2004). For example, COVIS predicts that delaying the feedback in verbal categorization should not affect performance (because the hypothesis-testing system relies on working memory) whereas delaying feedback in non-verbal categorization should impair learning (because the procedural learning system relies on dopamine-mediated reinforcement learning in the BG). This prediction was confirmed in Ashby et al. (2003). In addition, reducing dopamine levels in COVIS can account for many cognitive symptoms in PD patients such as perseveration, reduced sensitivity to negative feedback, and others (see Helie et al., 2012a, b). Likewise, dopamine elevation can account for the effect of positive affect on verbal category learning (Helie et al., 2012b). While most COVIS simulations have used a rate version of the model, a spiking version of the procedural-learning system has been used to account for some categorization results and extended to account for instrumental conditioning (Ashby and Crossley, 2011) and automaticity (Ashby et al., 2007).

As an alternative, Moustafa and Gluck (2011a,b) proposed a computational model of the striatum and prefrontal cortex that focuses on the dopamine projections to these areas as well as their interactions during multi-cue category learning. In this task, participants learn to select and pay attention to a single cue among a multi-cue pattern, and then make a categorization response. Participants learn this task via corrective feedback. In the model, the prefrontal cortex is essential for attentional selection while the striatum is used for motor response selection. Similar to COVIS, the Moustafa and Gluck (2011a,b) model can account for categorization deficits in PD patients by reducing dopamine levels in both the BG and prefrontal cortex, which is in agreement with empirical results (Kaasinen et al., 2001; Silberstein et al., 2005). Additionally, the Moustafa and Gluck (2011a,b) model can account for some effects of dopaminergic and anticholinergic medication. The Moustafa and Gluck (2011a,b) model assumes that the administration of anticholinergic medications in PD interferes with hippocampal function, which is also in agreement with empirical studies (Meco et al., 1984; Pondal et al., 1996; Ehrt et al., 2010; Herzallah et al., 2010). In contrast, the current version of COVIS has not been used to simulate medication effects in PD.

#### Synthesis

The reviewed models of categorization both agree that the BG, and its interaction with the prefrontal cortex, are essential for category learning. Furthermore, they agree that dopamine levels in both the BG and prefrontal cortex are important. While COVIS (Ashby et al., 1998) has been used to simulate a wider range of categorization tasks, the Moustafa and Gluck (2011a,b) model has been used to simulate more details in a smaller subset. For example, one limitation of the Moustafa and Gluck (2011a,b) model is that it does not simulate complex multi-cue learning tasks that involve paying attention to more than one stimulus (which can be done using COVIS). However, the Moustafa and Gluck (2011a,b) model can simulate the effect of dopaminergic medication, whereas COVIS has not been used to simulate medication effects. One important difference between the COVIS and Moustafa and Gluck (2011a,b) model is that COVIS assigns a different role for BG and cortical dopamine, namely error signal and signal gain (respectively). In contrast, Moustafa and Gluck (2011a,b) assign both of these roles to dopamine in both the BG and the prefrontal cortex. In addition, an important limitation of both models is that they oversimplify the anatomy of the BG by not including the indirect and hyperdirect pathways. Future work aimed at increasing the biological accuracy of COVIS and the Moustafa and Gluck (2011a,b) models may highlight some additional key differences between the modeling approaches and allow for selecting the most appropriate BG model of categorization.

### Instrumental conditioning

Instrumental conditioning (also called “operant” conditioning) is a process by which animals learn to behave in a way that will maximize reward and minimize punishment. In a typical instrumental conditioning experiment, a neutral environment is altered and begins generating rewards (acquisition phase). Next, the reward is removed from the environment and the environment reverts to its neutral state (extinction phase). Extinction is usually followed by a reacquisition phase, where the reward is introduced again in the same neutral environment. Typically, a new behavior is learned during the acquisition phase, and progressively disappears during the extinction phase. The behavior reappears during the reacquisition phase, usually at a much faster rate than during the initial acquisition phase. This phenomenon is called *fast reacquisition*. Much evidence implicates the BG in instrumental conditioning (e.g., O’Doherty et al., 2004; Yin et al., 2005), but the neurobiology underlying extinction and fast reacquisition is poorly understood.

#### Models

Ashby and Crossley (2011) proposed a spiking model of the direct pathway of the BG (see Figure 1) inspired by the COVIS procedural learning system (Ashby et al., 1998) to account for instrumental conditioning. The Ashby and Crossley (2011) model focuses on the TANs, a population of cholinergic interneurons in the striatum that is rarely included in CCN models of the BG. Pakhotin and Bracci (2007) have shown that TANs play an important role in inhibiting cortical activation of the MSNs (i.e., the projection neurons generally modeled in the direct and indirect pathways). As suggested by their name, TANs have a high baseline firing rate, but they learn to pause in rewarding contexts (Apicella, 2007). Ashby and Crossley (2011) suggest that one possible role for the TANs is to protect striatal learning from catastrophic interference and allow for fast reacquisition. In addition to the direct pathway, the Ashby and Crossley (2011) model includes a sensory association area, the supplementary motor area (SMA), and the CMPf complex.

The Ashby and Crossley (2011) model is an opened loop through the BG (from sensory association cortex to the SMA). The stimulus activates the sensory association cortex, which in turn activates the striatum and the direct pathway of the BG. At the same time, the context activates the CMPf complex, which transmits activation to the TANs (this pathway is not included in Figure 1). At the beginning of an experiment, the simulated subject does not know that the context is rewarding. Hence, the TANs do not pause, and the MSNs in the direct pathway cannot be activated by the sensory association cortex. This prevents stimulus—response association learning. During the acquisition phase, the TANs quickly learn that the context is rewarding and pause. The MSNs are thus released from inhibition and the model learns to produce the rewarding behavior using reinforcement learning. Next, during the extinction phase, the TANs learn that the context is no longer rewarding and stop pausing. This change inhibits the MSNs and interrupts cortico-striatal learning. Hence, the associations that were learned during the acquisition phase are protected. Finally, during the reacquisition phase, the context becomes rewarding again, and the TANs learn to pause. The MSNs are released from inhibition, and the learned associations are in the same state as in the acquisition phase, which produces fast reacquisition. Using this process, the model has been used to reproduce the acquisition, extinction, and fast reacquisition phases typical of instrumental conditioning and single-cell recording data from TANs showing that the cells learn to pause in rewarding contexts (Ashby and Crossley, 2011).

#### Synthesis

The Ashby and Crossley (2011) model is the only CCN model of instrumental conditioning that can simultaneously account for behavioral (e.g., fast reacquisition) and single-cell data (from the TANs). This model constitutes an important step in that it provides an implementation and numerical simulation of the theory that TANs learn to pause in rewarding contexts, and how this can affect reinforcement learning in the BG. However, the neuroanatomy of the BG was simplified in that only the direct pathway through one of the cortico-BG loops was included. It is unclear at this time how the TANs’ dynamics would affect the indirect pathway, or how the model would behave if more than one loops was included. Future work is needed to verify how the theory implemented in Ashby and Crossley (2011) behaves in a more detailed model of the BG.

### Probabilistic learning

Probabilistic learning typically refers to tasks where the association between the response and the reward is uncertain. Unlike most categorization experiments, the same response to the same stimulus can result in different outcomes on different trials. While probabilistic learning has been shown to rely on the BG since the mid-1990s (Knowlton et al., 1996), it took a decade before CCN models of the BG were used to attempt to account for probabilistic learning.

#### Models

The Frank (2005) model was originally proposed to account for cognitive deficits in parkinsonism. The model includes both the direct and indirect pathways through the BG (see Figure 1), the premotor cortex, and an unspecified input area (probably located in posterior cortex) (so the model is an opened loop). In the Frank (2005) model, the input activates both the premotor cortex and the striatum. However, cortical activation is insufficient to produce a response, so BG processing is required to gate the correct response. The focus of the model is on: (1) the role of the indirect pathway in probabilistic learning and (2) the role of dopamine in probabilistic learning. In the Frank (2005) model, the direct pathway is in charge of selecting the appropriate action (Go) whereas the indirect pathway is in charge of inhibiting inappropriate actions (NoGo). The direct and indirect pathways converge in the GPi and compete to control GPi activation, and eventually the response. Simulation results show that removing the indirect pathway in the model reduces performance, suggesting that both the direct and indirect pathways are essential in probabilistic learning. In addition, the effect of the indirect pathway needs to be specific to each action (so that the indirect pathway can individually inhibit each action).

As described in the neuroanatomy section above, the competition between the direct and indirect pathways is modulated by dopamine (the second focus of the Frank (2005) model). Specifically, higher dopamine levels increase activation in the direct pathway (e.g., through D1 receptors) and reduces activation in the indirect pathway (e.g., through D2 receptors). Hence, dopamine release following unexpected rewards results in long-term potentiation (LTP) in the direct pathway and long-term depression (LTD) in the indirect pathway. In contrast, dopamine dips following the unexpected absence of a reward reduces activation and produces LTD in the direct pathway but increases activation and produces LTP in the indirect pathway. The simulation results suggest that the dynamic range of the dopamine signal is crucial in probabilistic learning and reversal learning (e.g., when the response—reward associations are changed during learning). Reducing (to simulate PD) or increasing (to simulate medication overdose) dopamine levels can result in simulated Parkinsonian symptoms (Frank, 2005).

Another interesting model of probabilistic learning was recently proposed by Guthrie et al. (2013). The Guthrie et al. (2013) model is based on an earlier computational neuroscience model of the BG that focuses on the interaction between the direct and hyperdirect pathways (Leblois et al., 2006). The Guthrie et al. (2013) model includes two cortico-BG closed-loop that interact in the striatum. The first loop is called the “cognitive” loop and is used to identify the visual symbols used in the probabilistic learning task. The second loop is called the “motor” loop and is used to select a response based on the observed symbols. Some of the corticostriatal projections affect both loops, but the rest of the circuit is segregated. In both loops, the direct pathway is in charge of selecting the correct channel (i.e., identifying the symbols or the response) while the hyperdirect pathway sends non-specific inhibition to the GPi to produce a center-surround decision process. All corticostriatal synapses are plastic (using dopamine-mediated reinforcement learning) and the cognitive loop gradually learns to bias the motor loop, thus producing faster reaction times. The model successfully reproduces both neural firing rates and behavioral data in the double-arm bandit task.

The categorization models reviewed earlier have also been applied to probabilistic learning. The Moustafa and Gluck (2011a) model focused on the role of dopamine in probabilistic learning. In addition to simulating probabilistic learning with normal dopamine levels, Moustafa and Gluck (2011a) have simulated the effect of decreased dopamine (as in PD) and the effect of dopaminergic medication in both the BG and prefrontal cortex. The COVIS model has also been used to simulate probabilistic learning (Helie et al., 2012a). While COVIS was not used to simulate medication effects, the model could account for probabilistic learning with normal and reduced (as in PD) dopamine levels (with a dosage effect such that lowest levels of dopamine produced worst performance; see Knowlton et al., 1996).

#### Synthesis

The reviewed models of probabilistic learning tend to be more biologically detailed than the reviewed models of categorization. Specifically, the Frank (2005) model includes the direct and indirect pathways, whereas the Guthrie et al. (2013) model includes the direct and hyperdirect pathways. In contrast, COVIS (Ashby et al., 1998) and the Moustafa and Gluck (2011a,b) models only included the direct pathway. Interestingly, however, the Frank (2005) model does not include the same details as the Guthrie et al. (2013) model. Both models include the direct pathway for action selection, and use dopamine-mediated reinforcement learning to learn corticostriatal synapses. However, the Frank (2005) model uses the indirect pathway as a channel-specific excitatory process to cancel inappropriate actions whereas Guthrie et al. (2013) use the hyperdirect pathway as a non-specific excitatory process to cancel inappropriate actions. Neither model includes both the indirect and hyperdirect pathways. While there is agreement on the need for an excitatory process to enhance the contrast between the selected and non-selected actions, the exact process is still to be determined.

While the categorization models only included the direct pathway of the BG, one of their strengths is that, in addition to their generality, they also include other brain areas. For instance, Unlike the Frank (2005) model, the Moustafa and Gluck (2011a,b) model simulates the role of prefrontal cortex and its dopamine projections, which is in agreement with empirical studies (Mulder et al., 2003; Histed et al., 2009). Also, analysis of the parameter space in the COVIS simulations challenges the role of the BG for procedural learning in probabilistic learning, and suggests instead that the BG are used for hypothesis-testing in this task (Gluck et al., 2002). So, both categorization models agree on an important role for the prefrontal cortex in probabilistic learning, and this role is missing from both the Frank (2005) and the Guthrie et al. (2013) models. The most productive future approach might be to add a prefrontal cortex to the existing probabilistic learning models and see how this addition affects the dynamic of the different BG pathways.

### Working memory

Working memory is a cognitive function used to maintain and manipulate information in real-time for a short duration (seconds). While working memory has traditionally been associated with the prefrontal cortex (Fuster, 2008), Monchi et al. (2000) proposed that the BG may be required to maintain information in prefrontal cortex.

#### Models

The Monchi et al. (2000) model was originally proposed to account for working memory deficits in PD and schizophrenia. The model includes three BG-thalamocortical closed loops: two with the prefrontal cortex (one for spatial information and the other for object information), and one through the ACC (for strategy selection). In all three cases, only the direct pathway through the BG was included (see Figure 1). The role of the two prefrontal-BG loops is to maintain working memory information about the stimuli, whereas the ACC maintains the adopted strategy by inhibiting all the prefrontal cortex loops except one (i.e., representing the selected strategy). Visual input to the BG comes from the posterior parietal cortex (spatial) and inferior temporal cortex (object). The model output is located in the premotor cortex, and the nucleus accumbens (not shown in Figure 1) is used to provide feedback. In the model, the visual stimulus is input to the prefrontal cortex loops, and the stimulus activity is propagated through the direct pathway of the BG. As a result, the thalamus is released from GPi inhibition, and activation produced by the stimulus in the prefrontal cortex reverberates through closed-loops with the thalamus. When a response is required, the prefrontal cortex transfers its activation to the premotor cortex. If the response is incorrect, the nucleus accumbens sends a feedback signal to the ACC loop, which selects a new strategy by switching its inhibition to different prefrontal cortex loops. The Monchi et al. (2000) model has been used to simulate a delayed response task and the Wisconsin Card Sorting Test. Interestingly, reducing the connection strengths within the BG-thalamocortical loops produces Parkinsonian symptoms, whereas reducing nucleus accumbens activity produces deficits similar to those observed in schizophrenia (Monchi et al., 2000).

Five years later, Ashby et al. (2005) proposed the FROST model to account for intact spatial working memory maintenance. Similar to the Monchi et al. (2000) model, FROST includes the direct pathway of the BG (see Figure 1), and working memory maintenance relies on reverberating activation between the prefrontal cortex and the thalamus. However, unlike in the Monchi et al. (2000) model, only one prefrontal cortex loop is included, and thalamic activation is not sufficient to maintain prefrontal activity: a second set of closed-loops between the prefrontal cortex and posterior cortex needs to be simultaneously activated to maintain working memory information. In Ashby et al. (2005), the focus is on simulating spatial working memory tasks, and the area of posterior cortex required for working memory maintenance is the posterior parietal cortex. However, it is likely the case that the specific location in posterior cortex depends on what information is being maintained. For instance, if the items being maintained in working memory were objects, then it is likely that the posterior cortex area involved would be inferior temporal cortex. Another difference between FROST and the Monchi et al. (2000) model is that the striatum in FROST is activated by a different population of prefrontal neurons (separate from the working memory maintenance prefrontal population) whereas the same prefrontal neurons are used to activate the striatum and maintain information in Monchi et al. (2000). As a result, the striatum becomes activated only after the stimulus has disappeared in FROST, whereas the striatum becomes activated as soon as the stimulus appears in Monchi et al. (2000). These differences between FROST and Monchi et al. (2000) were motivated by recent single-cell recording results reviewed in Ashby et al. (2005). FROST has been used to reproduce single-cell recordings from many experiments in several brain regions, in addition to accounting for working memory capacity limitation and the relation between memory span and the ability to ignore distracting information (Ashby et al., 2005).

One common theme of the two previous models is that working memory activity is maintained by closed-loops between the thalamus and prefrontal cortex, and the main role of the BG is to release the thalamus from inhibition and allow for the reverberating activation to take place. However, this view was challenged by Frank et al. (2001) who proposed a model of BG-prefrontal cortex interaction in working memory. Specifically, Frank et al. (2001) argued that in order for the thalamus to contribute to working memory maintenance in the way described by the previous models, it would need to have a dedicated number of cells comparable to the number of cells dedicated to working memory in prefrontal cortex (which is unlikely). Instead, the Frank et al. (2001) model proposes that working memory maintenance is accomplished by reverberating loops between two cell populations within the prefrontal cortex. Similar to Monchi et al. (2000) and FROST (Ashby et al., 2005), the Frank et al. (2001) model includes the direct pathway through the BG (see Figure 1), but the role of the direct pathway is to “turn on the switch” on the prefrontal cortex cells and allow for reverberating activation. The “switch” can only be turned on if the prefrontal cortex cells from one population simultaneously receive activation from the BG and the other prefrontal cortex cell population. Once the switch is “on”, the BG is no longer required for working memory maintenance. The Frank et al. (2001) model has been used to simulate the 1-2-AX task, a working memory task that requires maintenance but also switching and selecting new items (Frank et al., 2001). Specifically, the 1-2-AX task requires the subject to maintain two cues in working memory in order to correctly select a response to a target sequence. The identity of the target sequence depends on the previous cue, which is used to trigger selection and switching.

One topic that was not addressed by any of the previous models of working memory is learning. How can the brain learn what is important, and what needs to be maintained in working memory? Moustafa and Maida (2007) proposed a computational model of prefrontal cortex and BG interactions that is similar to the Frank et al. (2001) model except that Moustafa and Maida (2007) also simulate: (a) temporal difference learning based on phasic dopamine signaling and (b) more than one corticostriatal loops that are responsible for both motor and cognitive processes. Specifically, the model includes a cortico-striatal motor loop and a cortico-striatal cognitive loop whose functions are action selection (choosing motor responses) and cognitive selection (choosing the perceptual information to be maintained in working memory), respectively. The model can account for the separate roles of the motor and cognitive loops in working memory maintenance, including delayed-response tasks.

Schroll et al. (2012) recently proposed a CCN model of working memory to address the problem of learning complex working memory tasks. The Schroll et al. (2012) model includes two separate BG-thalamocortical closed loops, one through the prefrontal cortex for maintenance and another through motor cortex to produce a response. Only the direct pathways were used for maintenance and response selection, but the hyperdirect pathway was also included in the prefrontal loop as a reset mechanism (see Figure 1). Specifically, visual information enters the model through the inferior temporal cortex, which then activates the lateral prefrontal cortex. This activation is transferred through the direct pathway of the BG and releases the thalamus from inhibition, which in turn activates the lateral prefrontal cortex. In the Schroll et al. (2012) model, working memory activation in the prefrontal cortex is maintained by a reverberating activation loop through the direct pathway, so the BG does not only act as a gating mechanism but is part of the maintenance loop (unlike Monchi et al., 2000; Frank et al., 2001; Ashby et al., 2005). At any moment, the prefrontal cortex can activate the STN, which increases activation in the GPi and interrupt working memory maintenance (i.e., the reset mechanism). More importantly, the connectivity between the prefrontal cortex and the striatum and the connections between the prefrontal cortex and the STN are learned using dopamine-mediated reinforcement learning. Hence, the model can automatically adapt and only maintain information that is rewarded in working memory. The model has been used to simulate a delayed response task, a delayed alternation task, and the 1-2-AX task (Schroll et al., 2012).

#### Synthesis

Working memory is one of the most active areas for CCN modeling of the BG. Five different models were reviewed, each having both commonalities and differences. First, all five models focused on the interaction between the BG and the prefrontal cortex, but only included the direct pathway of the BG for working memory maintenance and response selection. Hence, the neuroanatomy of the BG was not very detailed. Also, all models except Schroll et al. (2012) used the BG as a gating mechanism that turns working memory maintenance “on” or “off”. The main difference is that Monchi et al. (2000) and Ashby et al. (2005) used the BG to gate closed loops between the prefrontal cortex and the thalamus, whereas Frank et al. (2001) and Moustafa and Maida (2007) used the BG to gate closed loop between two populations of prefrontal cortex units. This differs from Schroll et al. (2012) where the BG was not used for gating, but instead was part of the working memory maintenance mechanism itself (i.e., the closed loop went through the BG). In all cases, however, working memory maintenance was achieved by closed loop through the prefrontal cortex.

Another important difference between the models is that the Ashby et al. (2005) and the Moustafa and Maida (2007) models focused on simple maintenance tasks. In contrast, the Monchi et al. (2000), Frank et al. (2001), and Schroll et al. (2012) models were able to simulate more complex tasks involving hierarchical structures and switching. Only the Moustafa and Maida (2007) and the Schroll et al. (2012) models include learning mechanisms that allowed for selecting the relevant information that needs to be maintained in working memory. The other models assumed a pre-filtering of the information.

Interestingly, there seems to be a progression and a building up of knowledge related to CCN models of working memory. The Schroll et al. (2012) model is the most recent, and also the most detailed. It is the only model that can learn and simulate complex tasks. However, this model departs from all the others in that the BG is not used as a gating mechanism but is part of the maintenance mechanism. This departure from previous modeling is not extensively discussed in Schroll et al. (2012), and it is unclear at this point what motivated this departure. More work is needed to determine which of these two roles the BG play in working memory, but the overlap in the models, and the progression in functionality, suggest a steady progress in CCN modeling of working memory.

### Sequence learning

Almost all our everyday behaviors and cognitive activities can be interpreted as a sequence of steps that bring us closer to achieving a goal. One key question is how can we learn to chain these sequences of substeps? Miyachi et al. (1997, 2002) have gathered much data suggesting that the BG is important in learning such sequences.

#### Models

Nakahara et al. (2001) formalized Miyachi et al. (1997, 2002) findings into a CCN model. According to Nakahara et al. (2001), sequences are learned in both visual and motor coordinates. The visual sequences are learned by a BG-thalamocortical closed-loop linking the anterior striatum with the dorsolateral prefrontal cortex while motor sequences are learned by a BG-thalamocortical closed-loop linking the posterior striatum with the SMA. Only the direct pathway through the BG is included in each loop (see Figure 1), and both the visual and motor loops learn every sequence in parallel using reinforcement learning. The visual loop learns faster than the motor loop, and response coordination between the loops is controlled by the pre-SMA. According to Nakahara et al. (2001), the visual loop relies on working memory and is important for rapid acquisition of sequences. However, the motor loop is more reliable and produces movement more rapidly after training. As a result, control is gradually transferred from the visual loop to the motor loop in the Nakahara et al. (2001) model. The Nakahara et al. (2001) model has been used to account for: (1) the time course of learning (including single-cell recordings and lesion studies); (2) the effect of opposite hand use; (3) the effect of sequence reversal; and (4) the change in brain locus from early to late sequence production (Nakahara et al., 2001).

#### Synthesis

The Nakahara et al. (2001) model is interesting for several reasons. First, it successfully accounts for lesion data, single-cell recordings, and behavioral phenomena. In addition, the transition from a visual loop to a motor loop represents an early attempt at bridging the gap between cognitive and motor functions of the BG. However, a recent study by Desmurget and Turner (2010) challenges the generality of the Nakahara et al. (2001) model. Specifically, Desmurget and Turner (2010) had monkeys perform a sequence of visually-cued joystick movements aimed at moving a cursor into a pre-determined part of a computer screen. After some training, muscimol was injected into the motor segment of the GPi to functionally disconnect the BG from the frontal cortex. The results show that the kinematics of the movements were impaired, but not sequence knowledge. Desmurget and Turner (2010) interpreted these results as suggesting that the BG contributes to motor execution in automatic sequence production, but not to the motor sequencing or the storage of the overlearned sequence. This result is problematic for the Nakahara et al. (2001) model.

### Automaticity

Automaticity results from overtraining in a task until performance requires little attentional resources and becomes highly rigid (Helie et al., 2010; Helie and Cousineau, 2011). Many computational models of automaticity development have assigned a role for the BG.

#### Models

First, in the Nakahara et al. (2001) model of sequence learning (above), automaticity in sequence production is characterized by a gradual transfer from the visual loop (which learns sequences in visual coordinates) to the motor loop (which learns sequences in motor coordinates). This corresponds well with the results of Miyachi et al. (1997, 2002), who showed using single-cell recordings that task-sensitive cells in early learning are mostly located in the anterior striatum whereas selective cells in late sequence production are mostly located in the posterior striatum (Miyachi et al., 2002). This specialization of the striatum is further supported by inactivation studies where muscimol (a GABA agonist) was injected in different parts of the striatum in early and late sequence production. Well-learned sequence production was selectively disrupted by muscimol injection in the middle-posterior putamen while early sequence production was selectively disrupted by muscimol injection in the anterior caudate and putamen (Miyachi et al., 1997).

However, a recent study by Desmurget and Turner (2010) challenges the generality of the Nakahara et al. (2001) model. Specifically, injecting muscimol into the motor segment of the GPi to functionally disconnect the BG from the frontal cortex affects the kinematics of the movements but not sequence knowledge. These results suggest that the BG contributes to motor execution in automatic sequence production, but not to the motor sequencing or the storage of the overlearned sequence. Interestingly, the results of Desmurget and Turner (2010) are consistent with a neurobiological model of automaticity in perceptual categorization (SPEED) (Ashby et al., 2007). SPEED uses the procedural system of COVIS (Ashby et al., 1998) (i.e., the direct pathway of an opened loop between posterior cortex and premotor areas) but also includes a Hebbian learning mechanism between posterior cortex and premotor areas. The role of the BG in SPEED is to learn to produce the correct categorization responses early in training to ensure that the correct motor plan in the premotor areas is consistently activated shortly after the visual representation in associative cortex (using dopamine-mediated reinforcement learning). This consistent association between associative and premotor cortical activity triggers Hebbian learning between associative cortex (stimulus) and the premotor areas (response), and the direct cortico-cortical connections eventually become strong enough so that the BG is no longer required to produce a response. When responding becomes purely cortical, the skill is said to be “automatic” [note that this is different from Nakahara et al. (2001), in which the posterior striatum is still required for automatic sequence production]. SPEED has been used to simulate single-cell recordings data in many categorization experiments, as well as human reaction times and accuracies in categorization (Ashby et al., 2007; Helie and Ashby, 2009).

While the Hikosaka et al. (2000) and SPEED models can account for how behavior becomes automatic, they cannot account for how automatic responses are overridden by goal-directed behavior when needed (e.g., when the stimulus—response associations change). Chersi et al. (2013) proposed a computational model of automaticity in instrumental conditioning that can account for the change back to goal-directed behavior when needed. The Chersi et al. (2013) model includes the prefrontal cortex (for goal representation), the motor cortex (for action representation), the sensory cortex (for stimulus representation), the BG (for action selection), and the thalamus (to relay information between the BG and the motor cortex). Two sets of connections are plastic: (1) connections from the prefrontal cortex to the motor cortex (to learn goal—response associations) and (2) connections from the sensory cortex to the striatum (to learn stimulus—response associations). According to this model, the stimulus activates the sensory cortex, which in turn activates a goal in prefrontal cortex and action representations in the striatum. For automatic behavior, the striatal activation propagates through both the direct and indirect pathways (see Figure 1) of the BG and an action is selected by inhibiting all but one action at the output level (SNr, but it is functionally equivalent to the GPi shown in Figure 1). The action that is not inhibited activates the appropriate response in motor cortex (through the thalamus). For goal-directed behaviors, the prefrontal activation propagates to the appropriate action in motor cortex. When an automatic action needs to be overwritten by a goal-directed behavior, the prefrontal cortex sends activation to the STN, which hyperpolarizes the SNr and prevents the BG from controlling the motor response (Chersi et al., 2013). The model has been successfully used to account for the development of automaticity in an instrumental conditioning task and the reversal of stimulus—response associations after automaticity had developed (Chersi et al., 2013).

#### Synthesis

The Nakahara et al. (2001) and the Chersi et al. (2013) models both assign the role of producing automatic behavior to the BG. However, this “classic” role of the BG in automaticity is difficult to reconcile with the Desmurget and Turner (2010) data. As an alternative, SPEED (Ashby et al., 2007) also assigns an important role to the BG in automaticity, but this role is restricted to training automatic cortico-cortical projections that can account for automaticity. Simply put, the BG is required to learn automatic behaviors, but the BG is no longer required to produce automatic behaviors once the cortico-cortical connectivity is sufficiently strong. The SPEED model can account for the Desmurget and Turner (2010) data, but it includes only the direct pathway of one loop through the BG. In contrast, the Nakahara et al. (2001) model includes two loops through the BG (only the direct pathways) and the Chersi et al. (2013) model includes both the direct and indirect pathways, but only one loop through the BG (similar to SPEED). In addition to being the most biologically detailed, the Chersi et al. (2013) model is the only reviewed model that can override automatic behavior using goal-directed behavior. This is an important function that was not accounted for by the previous models. However, like the Nakahara et al. (2001) model, the Chersi et al. (2013) model cannot account for the Desmurget and Turner (2010) data. To summarize, each one of these models was designed to account for a different aspect of automatic behavior, and successfully accounts for the aspect of automaticity for which it was designed. The next step is to explore how each one of these candidate models can account for the missing function/data that was the focus of the other candidate models.

## Motor function

This section describes motor functions that have been attributed to the BG and that have been simulated using CCN models. Hence, computational models that focus only on modeling biological data or motor function (e.g., kinematics) were not included. Similar to the section reviewing cognitive functions above, the model descriptions are conceptual, in that implementation details and formalities are not discussed. The reader is referred to the cited original papers for details and equations. Table 1 summarizes the reviewed models along with their respective components.

### Reaching

The BG has been implicated in reaching movements for many years (for a review, see Bischoff, 1998). Not surprisingly, PD patients show unmistakable changes in reaching movements, which can be used for diagnostic purposes (Brown and Jahanshahi, 1996). Simple reaching movements in PD patients show longer reaction times and movement times than normal controls. This reduced movement speed seen in PD reaching is called bradykinesia. From a physiological perspective, a typical reaching movement under normal conditions consists of a sequence of agonist-antagonist bursts. In contrast, a PD reaching movement is generally multi-staged and involves multiple agonist bursts. Furthermore, PD reaching movements have greater variability of hand position for larger movements (Sheridan and Flowers, 1990). PD patients also show impairment in sequential movements (Weiss et al., 1997). For example, during movements aimed at reaching a glass of water, PD patients exhibit an inordinately long pause between the reaching and retrieval of the glass.

#### Models

Several computational models relating dopamine deficiencies to impaired reaching movements have been proposed. One of the first models of PD reaching movements is the model of Bischoff (1998). Bischoff (1998) model includes the prefrontal cortex (for working memory/learning), the SMA (for sequence generation), the pre-SMA (for sequence preparation), motor cortex (for movement parameters), the thalamus (to filter information from the BG to cortex), and the BG. The BG model assigns complementary roles to the direct and indirect pathways (see Figure 1). According to Bischoff (1998), the role of the direct pathway is to inform the motor cortex of the movement’s next sensory state, while the role of the indirect pathway is to inhibit competing movements. The function of dopamine is to keep the balance between the two pathways, which is impaired in PD. The Bischoff (1998) model was used to simulate the reciprocal aiming task, a task where subjects are asked to alternatively tap a stylus between two targets as quickly as possible. Reducing the dopamine levels in the simulation reproduced bradykinesia and the exaggerated pauses in sequential movements observed in PD.

Magdoom et al. (2011) also proposed a model of the role of the BG in PD reaching movements. The model is cast in the framework of reinforcement learning and focuses on interactions between the motor cortex and the BG. The Magdoom et al. (2011) model uses the classical interpretations of BG pathways according to which the direct pathway facilitates movement, (i.e., the “Go” pathway), while the indirect pathway inhibits movements (i.e., the “NoGo” pathway). Switching between the two pathways is thought to be driven by striatal dopamine levels. However, Magdoom et al. (2011) also deviate from the classical “Go”/“NoGo” model of the BG by adding an intermediate regime called the *explore* regime. The explore regime is used to control the stochasticity of action selection when the gradient is absent or too weak to allow for reinforcement learning. The indirect pathway is proposed to be the substrate supporting the explore regime. Simulations show that under dopamine-deficient conditions of PD, the model spends less time in the “Go” regime while spending more time in the remaining two regimes. These regime changes were used to account for a variety of features of impaired reaching movements in PD including movement undershoot (Van Gemmert et al., 2003), bradykinesia, and increased path variability (Sheridan and Flowers, 1990).

#### Synthesis

Two models that highlight the role of the BG in reaching were reviewed. The Bischoff (1998) model includes, in addition to the BG, cortical areas such as prefrontal areas, the SMA and the pre-SMA. The model captures bradykinesia and abnormal pauses in sequential movements under Parkinsonian conditions. The reaching model of Magdoom et al. (2011) also incorporates the BG and the motor cortex. However, the Magdoom et al. (2011) model is cast in the framework of reinforcement learning, whereas there is no learning in the Bischoff (1998) model. Focus on learning makes the Magdoom et al. (2011) model consistent with the proposed role of BG in error correction (Lawrence, 2000). As a result, the Magdoom et al. (2011) model is more general and is consistent with the view that a wide range of BG functions can be explained within the framework of reinforcement learning (Chakravarthy et al., 2010). The compatibility of the Magdoom et al. (2011) model with other CCN models of BG function may facilitate integration to achieve a more complete model of BG function.

### Handwriting

Handwriting is a fine motor skill. PD patients often exhibit an impaired form of handwriting, known as micrographia, characterized by reduced letter size, a “kinky” handwriting contour, and abnormal fluctuations in velocity and acceleration (Teulings and Stelmach, 1991; Van Gemmert et al., 1999; Gangadhar et al., 2009). As a result, handwriting features like stroke size, peak acceleration, and stroke duration have been attributed to the BG and used for diagnosis of PD (Phillips et al., 1991; Van Gemmert et al., 2003).

#### Models

Although models of PD handwriting are scanty, extensive work has been done on modeling handwriting generation. One of the earliest insights into modeling handwriting consisted of performing a Fourier-like resolution of handwriting into oscillatory components (Hollerbach, 1981). This notion has led to the development of oscillatory or spiking neural network models of handwriting generation that can be trained to produce single characters (Schomaker, 1991; Kalveram, 1998). However, the models of Schomaker (1991) and Kalveram (1998) suffered from the absence of a plausible procedure for initializing the phases of neural oscillators, a difficulty that was solved in an oscillatory neural network model of handwriting generation proposed by Gangadhar et al. (2007).

While the above-described models did not explicitly include the BG, Gangadhar et al. (2008) combined the Gangadhar et al. (2007) handwriting generation model with a model of the BG. Similar to handwriting patterns observed in PD patients, the Gangadhar et al. (2008) model exhibits micrographia under conditions of reduced dopamine. Another significant feature of the model is the role of the dynamics of the STN and the GPe, which are connected as a loop to produce complex oscillations. Under pathological conditions, the oscillations of the STN and the GPe in the model are highly correlated, resembling the correlated neural firing from STN and GPe neurons under dopamine-deficient conditions observed in real electrophysiology experiments (Bergman et al., 1994; Brown et al., 2001). Under the influence of correlated oscillations of STN and GPe, the Gangadhar et al. (2008) model produces handwriting with large fluctuations in velocity in addition to diminutive letter size.

As another example, Contreras-Vidal and Stelmach (1995) attached a BG model to the VITE-WRITE model (Bullock et al., 1993) to simulate PD handwriting. The Contreras-Vidal and Stelmach (1995) model includes the direct, indirect, and hyperdirect pathways of the BG (see Figure 1), the SMA, and other motor and premotor areas. The role of the SMA is to read-in the next motor subprogram from the movement plan, while the role of the other motor and premotor areas is to produce the movement selected by the SMA. The role of the BG is to modulate the dynamics of the formation of movement trajectories (produced by VITE-WRITE). Reducing dopamine in the model to simulate PD results in reduced letter size, as observed in PD patients.

#### Synthesis

Two models of PD handwriting were reviewed above. The model of Contreras-Vidal and Stelmach (1995) combines the VITE-WRITE model (Bullock et al., 1993) with a BG model. The essence of the model consists of showing that dopamine reduction in PD causes an imbalance in the outputs of the direct and indirect pathways. Although constructed out of considerably different modeling components, the model of Gangadhar et al. (2008) also shows an imbalance in the activations of the direct and indirect pathways under simulated PD conditions, which causes a reduction in letter size. In addition, Gangadhar et al. (2008) also account for the oscillations in STN-GPe interaction. Loss of complexity in these oscillations under PD conditions were linked to higher velocity fluctuations and distorted handwriting contour in PD handwriting. To summarize, the indirect pathway appears to be critical in accounting for handwriting.

### Eye saccades

Eye saccades are rapid, darting eye movements that shift the fovea to points of interest in the visual scene. There is an extensive cortical and subcortical network that is responsible for saccade generation, and the BG play a key role among the subcortical substrates for saccade generation (Hikosaka et al., 2000). The influence of BG on saccades is propagated via the superior colliculus, a midbrain nucleus with a central role in saccade generation (not shown in Figure 1). Studies on Parkinsonian monkeys prepared by MPTP (a neurotoxin used to destroy dopaminergic neurons) infusion have observed prolonged saccades, longer reaction times, smaller peak velocities, and smaller amplitudes (Kato et al., 1995). Smaller peak velocities and smaller amplitudes in PD saccades may be comparable to bradykinesia and hypometria found in PD reaching movements. Similarly, analogous to PD tremor in extremities, some PD patients exhibit square-wave jerks in visually guided saccades (Rascol et al., 1991).

#### Models

Computational modeling literature that specifically focuses on the role of BG in saccade generation is rather limited. Dominey and Arbib (1992) proposed a model of the role of the BG in sequential saccade generation. Their model includes a number of relevant neural substrates such as the superior colliculus, thalamus, frontal eye fields, and the BG. In the Dominey and Arbib (1992) model, the BG is used as an indirect link between the frontal eye field and the superior colliculus, and its main function is to prevent saccades while a target stimulus is foveated. As such, only the direct pathway through the BG is modeled. The Dominey and Arbib (1992) model has been used to simulate simple saccade data, memory saccade data, double saccade data, compensatory saccade data, and lesion data.

Two decades later, Krishnan et al. (2011) proposed a model of the role of the BG in saccade generation using the principle of reinforcement learning. Similar to their model of PD reaching movement (Magdoom et al., 2011), the indirect pathway serves as an explorer that drives the saccades toward more rewarding targets. The model was able to successfully simulate two forms of visual search, namely feature search and conjunction search, a sequential saccade task, and a directional saccade task. When PD-related changes were incorporated in the model by reducing BG output, the model exhibited increased search times (Krishnan et al., 2011).

#### Synthesis

Two models of the role of BG in saccade generation were reviewed above (Dominey and Arbib, 1992; Krishnan et al., 2011). Both models can account for a range of saccade data in normal and lesioned/pathological conditions. The anatomical components incorporated by the two models are also quite similar. However, there are two main distinguishing features between the two models. One of these features is anatomical: the Dominey and Arbib (1992) model does not include the indirect pathway in the BG, whereas the indirect pathway plays a key role in the Krishnan et al. (2011) model. The second feature is functional: the Dominey and Arbib (1992) model does not involve learning, while that of Krishnan et al. (2011) model is based on reinforcement learning. These key differences make the Krishnan et al. (2011) model more biologically and functionally detailed.

## General discussion

This article presented a review of CCN models of cognitive and motor functions. The 19 reviewed models were organized to highlight BG functionality and classified according to six cognitive functions (i.e., categorization, instrumental conditioning, probabilistic learning, working memory, sequence learning, and automaticity) and three motor functions (i.e., reaching, handwriting, and visual saccades). On the one hand, some of the reviewed models are standalone models of specific functions of BG, e.g., the reaching model of Bischoff (1998). On the other hand, there are models that are specific instances of a more general learning framework applied to BG function. COVIS (Ashby et al., 1998, 2007; Ashby and Crossley, 2011), and the models of Chakravarthy and colleagues (Krishnan et al., 2011; Magdoom et al., 2011) belong to the second category. For example, both the models of Krishnan et al. (2011) and Magdoom et al. (2011) used a nearly identical reinforcement learning-based approach to account for the specific motor functions of reaching and saccade generation. A review article by Chakravarthy et al. (2010) proposes that an expanded framework based on reinforcement learning, adapted to BG anatomy and physiology, can be used to explain a wide variety of BG functions. Such a proposal needs a more extensive modeling and experimental investigation for further confirmation. However, interestingly, CCN models accounting for more than one function were accounting for more than one cognitive function or more than one motor function. None of the reviewed CCN models could account for at least one motor and one cognitive function simultaneously. This may be a serious limitation as behavioral experiments are beginning to reveal important interactions between motor and cognitive processes. Below, we discuss how cognitive processes might impact motor function, and point to novel directions for computational modeling studies.

### Interaction of motor and cognitive processes

While none of the models included simultaneously accounted cognitive and motor functions, they all had a cognitive and motor component. For example, the Ashby and Crossley (2011) model made a cognitive decision, but it also included premotor areas associated with the response. It just did not include a detailed model of the motor response (e.g., how is the left button pressed). Likewise, the Gangadhar et al. (2007) has to include a cognitive component specifying what character is to be drawn. However, the focus is on how the movement is produced. Perhaps the model that comes closest to integrating motor and cognitive functions is the model of Guthrie et al. (2013). In this model, both a cognitive and a motor decision are taken, and the interaction between these decisions is accounted for. However, this model does not include a detailed simulation of how the movement is produced. Therefore, it was only discussed in the context of cognitive function.

One way to explore how cognitive and motor functions interact is to explore disease states. For example, akinesia and bradykinesia in PD are arguably associated with BG (and corticostriatal circuits) dysfunction, while tremor is perhaps associated with cerebellar, thalamic, and STN abnormalities (Kassubek et al., 2002; Probst-Cousin et al., 2003; Weinberger et al., 2009; Zaidel et al., 2009; Mure et al., 2011). For example, Schillaci et al. (2011) found that PD patients with akinesia and rigidity as the predominant symptoms have significantly more widespread dopamine loss in the striatum than PD patients with tremor as the predominant symptom. Because these different brain areas (e.g., striatum, cerebellum) are also involved in different cognitive functions, it is reasonable to hypothesize that different PD motor symptoms may be associated with different cognitive impairments. Accordingly, Jankovic et al. (1990) found that PD patients with predominant tremor are less cognitively impaired than patients with bradykinesia. Below we explore some specific PD motor symptoms and their relation to cognition.

#### Akinesia

Experimental studies have shown that PD patients with severe akinesia are generally more cognitively impaired than PD patients with predominant tremor (Vakil and Herishanu-Naaman, 1998; Poletti et al., 2011, 2012; Poletti and Bonuccelli, 2013). For instance, PD patients with severe akinesia and rigidity symptoms are more impaired than PD patients with severe resting tremor at working memory tasks (Moustafa et al., 2013). Likewise, studies have shown that PD patients with tremor are usually less cognitively impaired than PD patients with akinesia or gait dysfunction (Burn et al., 2006; Lyros et al., 2008; Oh et al., 2009; Domellof et al., 2011). For example, Vakil and Herishanu-Naaman (1998) found that tremor-dominant PD patients are less impaired at procedural learning than akinesia-dominant PD patients.

Most motor models of the BG and corticostriatal circuit function have been able to explain the occurrence of akinesia and bradykinesia, but not tremor (Obeso et al., 2008). We suggest that motor performance may rely on cognitive processes in two different ways: (a) maintenance of motor plans in working memory while performing a sequence of movements, such as hand/leg movement, grasping, or reaching (Hayhoe et al., 2002; Ohbayashi et al., 2003; Piek et al., 2004; Issen and Knill, 2012); or (b) maintenance of goals in working memory while performing a motor act, such as maintaining the goal of grasping the cup in working memory while moving the hands (Batuev, 1989; McIntyre et al., 1998; Matsumoto et al., 2003). This relationship between cognitive and motor processes could explain why some cognitive training programs are effective at treating motor dysfunction in PD patients (Disbrow et al., 2012). Although this is speculative, computational models are needed to explicitly study the complex relationship between motor and cognitive processes in healthy subjects and PD patients.

#### Freezing of gait

Freezing of gait—paroxysmal cessation of motor output—is a common symptom in advanced PD (Hoehn and Yahr stage 2+) (Giladi et al., 1992). Freezing of gait is debilitating since it often leads to falls and, importantly, is not manageable by common psychopharmacological medications (Giladi et al., 1992; Matar et al., 2013).

Research shows that perceptual and cognitive factors play a role in successful locomotion and the occurrence of freezing of gait episodes in PD patients (Lewis and Barker, 2009; Naismith et al., 2010; Matar et al., 2013). For example, providing auditory or visual cues or instructions can often reduce the occurrence of freezing behavior in PD patients (Lewis and Barker, 2009). Other studies found that walking dysfunction in PD is related to difficulty in resolving response interference produced by distractors (Plotnik et al., 2011; Vandenbossche et al., 2011). Locomotive dysfunction in PD is associated with brain volume changes (Kostic et al., 2012; Tessitore et al., 2012) and aberrant neural activity within the prefrontal cortex (Matsui et al., 2005; Shine et al., 2013), suggesting a role for cognitive processes in locomotion.

There are currently no computational models that simulate the role of cognitive processes in the occurrence of freezing of gait in PD patients. Prior computational models of BG-cortex interactions have focused on the simulation of cognitive processes (O’Reilly and Frank, 2006), learning, or simple action selection in static environments (Gurney et al., 2001) without considering how cognitive factors might affect motor actions such as locomotion. Future models should simulate how the cortex represents multiple inputs (including perceptual and cognitive) that feed into the BG, which is important for action selection (e.g., move right, left, forward, etc.). Future models should also be more dynamical in that they should continuously receive and update perceptual input from the environment and produce motor output (step right, left, …), which then takes the model to a new perceptual input, and so forth.

### What is the role of the basal ganglia in cognitive and motor function?

In addition to the current unavailability of CCN models of the BG that can simultaneously account for cognitive and motor function, another limitation of the current state of BG modeling is the absence of consensus on the specific function of the BG in a given task. For example, many CCN models of working memory assign a role for the BG, but some models use the BG as a gating mechanism allowing for thalamo-cortical loops (e.g., Monchi et al., 2000; Ashby et al., 2005), while others use the BG as a gating mechanism for cortico-cortical loops (e.g., Frank et al., 2001) or as the actual maintenance mechanism (Schroll et al., 2012). As with many other cognitive and motor functions, CCN models are critical in pinpointing the specific function of the BG in the cognitive task (e.g., working memory). Computational models can be simulated to identify the consequences of different design choices, and these predictions need to be tested empirically. While models tend to do very well at simulating the function that motivated the model, it is unclear at this point how the model can handle other (different) functions. One way to select useful BG CCN models is to consider generalization capabilities. Towards this end, general integrative frameworks are most useful. For example, the reinforcement learning approach of Chakravarthy and colleagues (Krishnan et al., 2011; Magdoom et al., 2011) or the COVIS-based approach of Ashby and colleagues (Ashby et al., 1998; Apicella, 2007; Ashby and Crossley, 2011) are useful because they have been used to account for functions that were outside of the original scope of the model. Other models of cognitive and motor functions need to be generalized to account for data for which they were not originally designed to build biological “cognitive architectures”. Frameworks that are already general should attempt to bridge the gap between CCN models of cognitive function and CCN models of motor function. This could be achieved by integrating existing models. For example, a detailed CCN model of motor function could be added to the COVIS framework. Likewise, a detailed CCN model of cognitive function could be added to the reinforcement-learning-based approach of Chakravarthy and colleagues. While more data will help in eliminating some of the candidate CCN BG models, generalization and integration will be required to avoid overfitting the model to the available data.

## Conflict of interest statement

The authors declare that the research was conducted in the absence of any commercial or financial relationships that could be construed as a potential conflict of interest.

## References

[B1] Apicella P. (2007). Leading tonically active neurons of the striatum from reward detection to context recognition. Trends Neurosci. 30, 299–306 10.1016/j.tins.2007.03.011 17420057

[B2] Ashby F. G. Alfonso-Reese L. A. Turken A. U. Waldron E. M. (1998). A neuropsychological theory of multiple systems in category learning. Psychol. Rev. 105, 442–481 10.1037//0033-295x.105.3.442 9697427

[B3] Ashby F. G. Crossley M. J. (2011). A computational model of how cholinergic interneurons protect striatal-dependent learning. J. Cogn. Neurosci. 23, 1549–1566 10.1162/jocn.2010.21523 20521851

[B4] Ashby F. G. Ell S. W. Valentin V. V. Casale M. B. (2005). FROST: a distributed neurocomputational model of working memory maintenance. J. Cogn. Neurosci. 17, 1728–1743 10.1162/089892905774589271 16269109

[B5] Ashby F. G. Ell S. W. Waldron E. M. (2003). Procedural learning in perceptual categorization. Mem. Cognit. 31, 1114–1125 10.3758/bf03196132 14704026

[B6] Ashby F. G. Ennis J. M. Spiering B. J. (2007). A neurobiological theory of automaticity in perceptual categorization. Psychol. Rev. 114, 632–656 10.1037/0033-295x.114.3.632 17638499

[B7] Ashby F. G. Gott R. E. (1988). Decision rules in the perception and categorization of multidimensional stimuli. J. Exp. Psychol. Learn. Mem. Cogn. 14, 33–53 10.1037/0278-7393.14.1.33 2963894

[B8] Ashby F. G. Helie S. (2011). A tutorial on computational cognitive neuroscience: modeling the neurodynamics of cognition. J. Math. Psychol. 55, 273–289 10.1016/j.jmp.2011.04.003 21841845 PMC3153062

[B9] Assadi S. M. Yucel M. Pantelis C. (2009). Dopamine modulates neural networks involved in effort-based decision-making. Neurosci. Biobehav. Rev. 33, 383–393 10.1016/j.neubiorev.2008.10.010 19046987

[B10] Batuev A. S. (1989). Neuronal mechanisms of goal-directed behaviour in monkeys. Int. J. Neurosci. 44, 59–66 10.3109/00207458908986183 2518573

[B11] Bergman H. Whichman T. Karmon B. DeLong M. R. (1994). The primate subthalamic nucleus. II. neural activity in MPTP model of Parkinsonism. J. Neurophysiol. 72, 507–520 7983515 10.1152/jn.1994.72.2.507

[B12] Bernheimer H. Birkmayer W. Hornykiewicz O. Jellinger K. Seitelberger F. (1973). Brain dopamine and the syndromes of Parkinson and huntington clinical, morphological and neurochemical correlations. J. Neurol. Sci. 20, 415–455 10.1016/0022-510x(73)90175-5 4272516

[B13] Bischoff A. (1998). Modeling the Basal Ganglia in the Control of Arm Movements. Doctoral Dissertation, University of Southern California

[B14] Brown P. Olivero A. Mazzone P. Insola A. Tonali P. Lazzaro V. D. (2001). Dopamine dependency of oscillations in between subthalamic neucleus and pallidum in Parkinson’s disease. J. Neurosci. 21, 1033–1038 11157088 10.1523/JNEUROSCI.21-03-01033.2001 PMC6762327

[B15] Brown R. G. Jahanshahi M. (1996). Cognitive-motor function in Parkinson’s disease. Eur. Neurol. 36, 24–31 10.1159/000118880 8791018

[B16] Bullock D. Grossberg S. Mannes C. (1993). A neural network model for cursive script production. Biol. Cybern. 70, 15–28 10.1007/bf00202562

[B17] Burn D. J. Rowan E. N. Allan L. M. Molloy S. O’Brien J. T. McKeith I. G. (2006). Motor subtype and cognitive decline in Parkinson’s disease, Parkinson’s disease with dementia and dementia with Lewy bodies. J. Neurol. Neurosurg. Psychiatry 77, 585–589 10.1136/jnnp.2005.081711 16614017 PMC2117449

[B18] Calzavara R. Mailly P. Haber S. N. (2007). Relationship between the corticostriatal terminals from areas 9 and 46 and those from area 8A, dorsal and rostral premotor cortex and area 24c: an anatomical substrate for cognition to action. Eur. J. Neurosci. 26, 2005–2024 10.1111/j.1460-9568.2007.05825.x 17892479 PMC2121143

[B19] Chakravarthy V. Joseph D. Bapi R. S. (2010). What do the basal ganglia do? A modeling perspective. Biol. Cybern. 103, 237–253 10.1007/s00422-010-0401-y 20644953

[B20] Chersi F. Mirolli M. Pezzulo G. Baldassarre G. (2013). A spiking model of the cortico-basal ganglia circuits for goal-directed and habitual action learning. Neural Netw. 41, 212–224 10.1016/j.neunet.2012.11.009 23266482

[B21] Contreras-Vidal J. Stelmach G. (1995). A neural model of basal ganglia-thalamocortical relations in normal and Parkinsonian movement. Biol. Cybern. 73, 467–476 10.1007/bf00201481 7578481

[B22] Desmurget M. Turner R. S. (2010). Motor sequences and the basal ganglia: kinematics, not habits. J. Neurosci. 30, 7685–7690 10.1523/jneurosci.0163-10.2010 20519543 PMC2906391

[B23] Disbrow E. A. Russo K. A. Higginson C. I. Yund E. W. Ventura M. I. Zhang L. (2012). Efficacy of tailored computer-based neurorehabilitation for improvement of movement initiation in Parkinson’s disease. Brain Res. 1452, 151–164 10.1016/j.brainres.2012.02.073 22459048

[B24] Domellof M. E. Elgh E. Forsgren L. (2011). The relation between cognition and motor dysfunction in drug-naive newly diagnosed patients with Parkinson’s disease. Mov. Disord. 26, 2183–2189 10.1002/mds.23814 21661051

[B25] Dominey P. F. Arbib M. A. (1992). A cortico-subcortical model for generation of spatially accurate sequential saccades. Cereb. Cortex 2, 153–175 10.1093/cercor/2.2.153 1633413

[B26] Dreher J. C. Burnod Y. (2002). An integrative theory of the phasic and tonic modes of dopamine modulation in the prefrontal cortex. Neural Netw. 15, 583–602 10.1016/s0893-6080(02)00051-5 12371514

[B27] Durstewitz D. Seamans J. K. (2008). The dual-state theory of prefrontal cortex dopamine function with relevance to catechol-o-methyltransferase genotypes and schizophrenia. Biol. Psychiatry 64, 739–749 10.1016/j.biopsych.2008.05.015 18620336

[B28] Ehrt U. Broich K. Larsen J. P. Ballard C. Aarsland D. (2010). Use of drugs with anticholinergic effect and impact on cognition in Parkinson’s disease: a cohort study. J. Neurol. Neurosurg. Psychiatry 81, 160–165 10.1136/jnnp.2009.186239 19770163

[B29] Fearnley J. M. Lees A. J. (1991). Ageing and Parkinson’s disease: substantia nigra regional selectivity. Brain 114, 2283–2301 10.1093/brain/114.5.2283 1933245

[B30] Flaherty A. W. Graybiel A. M. (1994). Input-output organization of the sensorimotor striatum in the squirrel monkey. J. Neurosci. 14, 599–610 7507981 10.1523/JNEUROSCI.14-02-00599.1994 PMC6576827

[B31] Frank M. J. (2005). Dynamic dopamine modulation in the basal ganglia: a neurocomputational account of cognitive deficits in medicated and nonmedicated Parkinsonism. J. Cogn. Neurosci. 17, 51–72 10.1162/0898929052880093 15701239

[B32] Frank M. J. Loughry B. O’Reilly R. C. (2001). Interactions between frontal cortex and basal ganglia in working memory: a computational model. Cogn. Affect. Behav. Neurosci. 1, 137–160 10.3758/cabn.1.2.137 12467110

[B33] Fuster J. M. (2008). The Prefrontal Cortex. 4th Edn Singapore: Academic Press

[B34] Gangadhar G. Joseph D. Chakravarthy V. S. (2007). An oscillatory neuromotor model of handwriting generation. Int. J. Document Anal. Recognition 10, 69–84 10.1007/s10032-007-0046-0

[B35] Gangadhar G. Joseph D. Chakravarthy V. S. (2008). Understanding Parkinsonian handwriting using a computational model of basal ganglia. Neural Comput. 20, 2491–2525 10.1162/neco.2008.03-07-498 18386983

[B36] Gangadhar G. Joseph D. Srinivasan A. V. Subramanian D. Keshavan S. Chakravarthy V. S. (2009). A computational model of Parkinsonian handwriting that high lights the role of the indirect pathway in the basal ganglia. Hum. Mov. Sci. 28, 602–618 10.1016/j.humov.2009.07.008 19720411

[B37] Gerfen C. R. Engber T. M. Mahan L. C. Susel Z. Chase T. N. Monsma F. J. (1990). D1 and D2 dopamine receptor-regulated gene expression of striatonigral and striatopallidal neurons. Science 250, 1429–1432 10.1126/science.2147780 2147780

[B38] Gerfen C. R. Young W. S. (1988). Distribution of striatonigral and striatopallidal peptidergic neurons in both patch and matrix compartments: an in situ hybridization histochemistry and fluorescent retrograde tracing study. Brain Res. 460, 161–167 10.1016/0006-8993(88)91217-6 2464402

[B39] Gibb W. R. Lees A. J. (1991). Anatomy, pigmentation, ventral and dorsal subpopulations of the substantia nigra and differential cell death in Parkinson’s disease. J. Neurol. Neurosurg. Psychiatry 54, 388–396 10.1136/jnnp.54.5.388 1865199 PMC488535

[B40] Giladi N. McMahon D. Przedborski S. Flaster E. Guillory S. Kostic V. (1992). Motor blocks in Parkinson’s disease. Neurology 42, 333–339 10.1212/WNL.42.2.333 1736161

[B41] Gluck M. A. Shohamy D. Myers C. (2002). How do people solve the “Weather Prediction” Task?: individual variability in strategies for probabilistic category learning. Learn. Mem. 9, 408–418 10.1101/lm.45202 12464701 PMC187590

[B42] Gotham A. M. Brown R. G. Marsden C. D. (1988). “Frontal” cognitive function in patients with Parkinson’s disease “on”and “off” levodopa. Brain 111, 299–321 10.1093/brain/111.2.299 3378138

[B43] Gurney K. Prescott T. J. Redgrave P. (2001). A computational model of action selection in the basal ganglia. II. Analysis and simulation of behaviour. Biol. Cybern. 84, 411–423 10.1007/pl00007985 11417053

[B44] Guthrie M. Leblois A. Garenne A. Boraud T. (2013). Interaction between cognitive and motor cortico-basal ganglia loops during decision making: a computational study. J. Neurophysiol. 109, 3025–3040 10.1152/jn.00026.2013 23536713

[B45] Haber S. N. Kim K. S. Mailly P. Calzavara R. (2006). Reward-related cortical inputs define a large striatal region in primates that interface with associative cortical connections, providing a substrate for incentive-based learning. J. Neurosci. 26, 8368–8376 10.1523/jneurosci.0271-06.2006 16899732 PMC6673798

[B46] Hayhoe M. Aivar P. Shrivastavah A. Mruczek R. (2002). Visual short-term memory and motor planning. Prog. Brain Res. 140, 349–363 10.1016/s0079-6123(02)40062-3 12508602

[B47] Helie S. Ashby G. F. (2009). “A neurocomputational model of automaticity and maintenance of abstract rules,” in Proceedings of the International Joint Conference on Neural Networks (IEEE Press), 1192–1198

[B48] Helie S. Cousineau D. (2011). The cognitive neuroscience of automaticity: behavioral and brain signatures. Cogn. Sci. 6, 25–43

[B49] Helie S. Paul E. J. Ashby F. G. (2012a). A neurocomputational account of cognitive deficits in Parkinson’s disease. Neuropsychologia 50, 2290–2302 10.1016/j.neuropsychologia.2012.05.033 22683450 PMC4220550

[B50] Helie S. Paul E. J. Ashby F. G. (2012b). Simulating the effect of dopamine imbalance on cognition: from positive affect to Parkinson’s disease. Neural. Netw. 32, 74–85 10.1016/j.neunet.2012.02.033 22402326 PMC3368085

[B51] Helie S. Waldschmidt J. G. Ashby F. G. (2010). Automaticity in rule-based and information-integration categorization. Atten. Percept. Psychophys. 72, 1013–1031 10.3758/app.72.4.1013 20436197

[B52] Herzallah M. M. Moustafa A. A. Misk A. J. Al-Dweib L. H. Abdelrazeq S. A. Myers C. E. (2010). Depression impairs learning whereas anticholinergics impair transfer generalization in Parkinson patients tested on dopaminergic medications. Cogn. Behav. Neurol. 23, 98–105 10.1097/wnn.0b013e3181df3048 20535058

[B53] Hikosaka O. Takikawa Y. Kawagoe R. (2000). Role of the basal ganglia in the control of purposive saccadic eye movements. Physiol. Rev. 80, 953–978 10893428 10.1152/physrev.2000.80.3.953

[B54] Histed M. H. Pasupathy A. Miller E. K. (2009). Learning substrates in the primate prefrontal cortex and striatum: sustained activity related to successful actions. Neuron 63, 244–253 10.1016/j.neuron.2009.06.019 19640482 PMC2874751

[B55] Hollerbach J. M. (1981). An oscillation theory of handwriting. Biol. Cybern. 39, 139–156 10.1007/bf00336740

[B56] Issen L. A. Knill D. C. (2012). Decoupling eye and hand movement control: visual short-term memory influences reach planning more than saccade planning. J. Vis. 12, 1–13 10.1167/12.1.3 22219310

[B57] Jankovic J. McDermott M. Carter J. Gauthier S. Goetz C. Golbe L. (1990). Variable expression of Parkinson’s disease: a base-line analysis of the DATATOP cohort. Neurology 40, 1529–1534 10.1212/wnl.40.10.1529 2215943

[B58] Kaasinen V. Nurmi E. Bergman J. Eskola O. Solin O. Sonninen P. (2001). Personality traits and brain dopaminergic function in Parkinson’s disease. Proc. Natl. Acad. Sci. U S A 98, 13272–13277 10.1073/pnas.231313198 11687621 PMC60860

[B59] Kalveram K. T. (1998). “A neural oscillator model learning given trajectories, or how an allo-imitation algorithm can be implemented into a motor controller,” in Motor Behavior and Human Skill: A Multi-Disciplinary Approach, ed Piek J. (Champaign, IL, USA: Human Kinetics Publishers), 127–140

[B60] Kassubek J. Juengling F. D. Hellwig B. Spreer J. Lucking C. H. (2002). Thalamic gray matter changes in unilateral Parkinsonian resting tremor: a voxel-based morphometric analysis of 3-dimensional magnetic resonance imaging. Neurosci. Lett. 323, 29–32 10.1016/s0304-3940(02)00111-8 11911983

[B61] Kato M. Miyashita N. Hikosaka O. Matsumura M. Usui S. Kori A. (1995). Eye movements in monkeys with local dopamine depletion in caudate nucleus.I. Deficits in spontaneous saccades. J. Neurosci. 15, 912–927 7823189 10.1523/JNEUROSCI.15-01-00912.1995 PMC6578295

[B62] Kemp J. M. Powell T. P. S. (1970). The cortico-striate projections in the monkey. Brain 93, 525–546 10.1093/brain/93.3.525 4990231

[B63] Kincaid A. E. Zheng T. Wilson C. J. (1998). Connectivity and convergence of single corticostriatal axons. J. Neurosci. 18, 4722–4731 9614246 10.1523/JNEUROSCI.18-12-04722.1998 PMC6792707

[B64] Knowlton B. J. Mangels J. A. Squire L. R. (1996). A neostriatal habit learning system in humans. Science 273, 1399–1402 10.1126/science.273.5280.1399 8703077

[B65] Kostic V. S. Agosta F. Pievani M. Stefanova E. Jecmenica-Lukic M. Scarale A. (2012). Pattern of brain tissue loss associated with freezing of gait in Parkinson disease. Neurology 78, 409–416 10.1212/wnl.0b013e318245d23c 22282641

[B66] Krishnan R. Ratnadurai S. Subramanian D. Chakravarthy V. S. Rengaswamy M. (2011). Modeling the role of basal ganglia in saccade generation: is the indirect pathway the explorer? Neural Netw. 24, 801–813 10.1016/j.neunet.2011.06.002 21726978

[B67] Lawrence A. D. (2000). Error correction and the basal ganglia: similar computations for action, cognition and emotion? Trends Cogn. Sci. 4, 365–367 10.1016/s1364-6613(00)01535-7 11025274

[B68] Leblois A. Boraud T. Meissner W. Bergman H. Hansel D. (2006). Competition between feedback loops underlies normal and pathological dynamics in the basal ganglia. J. Neurosci. 26, 3567–3583 10.1523/jneurosci.5050-05.2006 16571765 PMC6673853

[B69] Lewis S. J. Barker R. A. (2009). A pathophysiological model of freezing of gait in Parkinson’s disease. Parkinsonism Relat. Disord. 15, 333–338 10.1016/j.parkreldis.2008.08.006 18930430

[B70] Lyros E. Messinis L. Papathanasopoulos P. (2008). Does motor subtype influence neurocognitive performance in Parkinson’s disease without dementia? Eur. J. Neurol. 15, 262–267 10.1111/j.1468-1331.2007.02046.x 18190508

[B71] Maddox W. T. Ashby F. G. (2004). Dissociating explicit and procedural-learning based systems of perceptual category learning. Behav. Processes 66, 309–332 10.1016/j.beproc.2004.03.011 15157979

[B72] Magdoom M. Subramanian D. Chakravarthy V. S. Ravindran B. Amari S. Meenakshisundaram N. (2011). Modeling basal ganglia for understanding Parkinsonian reaching movements. Neural Comput. 23, 477–516 10.1162/neco_a_00073 21105828

[B73] Matar E. Shine J. M. Naismith S. L. Lewis S. J. (2013). Using virtual reality to explore the role of conflict resolution and environmental salience in freezing of gait in Parkinson’s disease. Parkinsonism Relat. Disord. 19, 937–942 10.1016/j.parkreldis.2013.06.002 23831480

[B74] Matsui H. Udaka F. Miyoshi T. Hara N. Tamaura A. Oda M. (2005). Three-dimensional stereotactic surface projection study of freezing of gait and brain perfusion image in Parkinson’s disease. Mov. Disord. 20, 1272–1277 10.1002/mds.20520 16007622

[B75] Matsumoto K. Suzuki W. Tanaka K. (2003). Neuronal correlates of goal-based motor selection in the prefrontal cortex. Science 301, 229–232 10.1126/science.1084204 12855813

[B76] McIntyre J. Stratta F. Lacquaniti F. (1998). Short-term memory for reaching to visual targets: psychophysical evidence for body-centered reference frames. J. Neurosci. 18, 8423–8435 9763485 10.1523/JNEUROSCI.18-20-08423.1998 PMC6792850

[B77] Meco G. Casacchia M. Lazzari R. Franzese A. Castellana F. Carta A. (1984). Mental impairment in Parkinson’s disease. The role of anticholinergic drugs. Acta Psychiatr. Belg. 84, 325–335 6507124

[B78] Miyachi S. Hikosaka O. Lu X. (2002). Differential activation of monkey striatal neurons in the early and late stages of procedural learning. Exp. Brain Res. 146, 122–126 10.1007/s00221-002-1213-7 12192586

[B79] Miyachi S. Hikosaka O. Miyashita K. Kárádi Z. Rand M. K. (1997). Differential roles of monkey striatum in learning of sequential hand movement. Exp. Brain Res. 115, 1–5 10.1007/pl00005669 9224828

[B80] Monchi O. Taylor J. G. Dagher A. (2000). A neural model of working memory processes in normal subjects, Parkinson’s disease and schizophrenia for fMRI design and predictions. Neural Netw. 13, 953–973 10.1016/s0893-6080(00)00058-7 11156204

[B81] Moustafa A. A. Bell P. Eissa A. M. Hewedi D. H. (2013). The effects of clinical motor variables and medication dosage on working memory in Parkinson’s disease. Brain Cogn. 82, 137–145 10.1016/j.bandc.2013.04.001 23660434

[B82] Moustafa A. A. Cohen M. X. Sherman S. J. Frank M. J. (2008). A role for dopamine in temporal decision making and reward maximization in Parkinsonism. J. Neurosci. 28, 12294–12304 10.1523/jneurosci.3116-08.2008 19020023 PMC3049941

[B83] Moustafa A. A. Gluck M. A. (2011a). A neurocomputational model of dopamine and prefrontal-striatal interactions during multi-cue category learning by Parkinson’s patients. J. Cogn. Neurosci. 23, 151–167 10.1162/jocn.2010.21420 20044893

[B84] Moustafa A. A. Gluck M. A. (2011b). Computational cognitive models of prefrontal-striatal-hippocampal interactions in Parkinson’s disease and schizophrenia. Neural Netw. 24, 575–591 10.1016/j.neunet.2011.02.006 21411277

[B85] Moustafa Herzallah M. M. Gluck M. A. (2013). Dissociating the cognitive effects of levodopa versus dopamine agonists in a neurocomputational model of learning in Parkinson’s disease. Neurodegener. Dis. 11, 102–111 10.1159/000341999 23128796

[B86] Moustafa A. A. Maida A. S. (2007). Using TD learning to simulate working memory performance in a model of the prefrontal cortex and basal ganglia. Cogn. Syst. Res. 8, 262–281 10.1016/j.cogsys.2007.02.001

[B87] Mulder A. B. Nordquist R. E. Orgut O. Pennartz C. M. (2003). Learning-related changes in response patterns of prefrontal neurons during instrumental conditioning. Behav. Brain Res. 146, 77–88 10.1016/j.bbr.2003.09.016 14643461

[B88] Mure H. Hirano S. Tang C. C. Isaias I. U. Antonini A. Ma Y. (2011). Parkinson’s disease tremor-related metabolic network: characterization, progression and treatment effects. Neuroimage 54, 1244–1253 10.1016/j.neuroimage.2010.09.028 20851193 PMC2997135

[B89] Naismith S. L. Shine J. M. Lewis S. J. (2010). The specific contributions of set-shifting to freezing of gait in Parkinson’s disease. Mov. Disord. 25, 1000–1004 10.1002/mds.23005 20198644

[B90] Nakahara H. Doya K. Hikosaka O. (2001). Parallel cortico-basal ganglia mechanisms for acquisition and execution of visuomotor sequences-a computational approach. J. Cogn. Neurosci. 13, 626–647 10.1162/089892901750363208 11506661

[B91] Nambu A. Tokuno H. Takada M. (2002). Functional significance of the cortico-subthalamo-pallidal ‘hyperdirect’ pathway. Neurosci. Res. 43, 111–117 10.1016/s0168-0102(02)00027-5 12067746

[B92] Niv Y. Daw N. D. Joel D. Dayan P. (2007). Tonic dopamine: opportunity costs and the control of response vigor. Psychopharmacology (Berl) 191, 507–520 10.1007/s00213-006-0502-4 17031711

[B93] Obeso J. A. Marin C. Rodriguez-Oroz C. Blesa J. Benitez-Temino B. Mena-Segovia J. (2008). The basal ganglia in Parkinson’s disease: current concepts and unexplained observations. Ann. Neurol. 64, S30–S46 10.1002/ana.21481 19127584

[B94] O’Doherty J. Dayan P. Schultz J. Deichmann R. Friston K. Dolan R. J. (2004). Dissociable roles of ventral and dorsal striatum in instrumental conditioning. Science 304, 452–454 10.1126/science.1094285 15087550

[B95] Oh J. Y. Kim Y. S. Choi B. H. Sohn E. H. Lee A. Y. (2009). Relationship between clinical phenotypes and cognitive impairment in Parkinson’s disease (PD). Arch. Gerontol. Geriatr. 49, 351–354 10.1016/j.archger.2008.11.013 19136159

[B96] Ohbayashi M. Ohki K. Miyashita Y. (2003). Conversion of working memory to motor sequence in the monkey premotor cortex. Science 301, 233–236 10.1126/science.1084884 12855814

[B97] O’Reilly R. C. Frank M. J. (2006). Making working memory work: a computational model of learning in the prefrontal cortex and basal ganglia. Neural Comput. 18, 283–328 10.1162/089976606775093909 16378516

[B98] Pakhotin P. Bracci E. (2007). Cholinergic interneurons control the excitatory input to the striatum. J. Neurosci. 27, 391–400 10.1523/jneurosci.3709-06.2007 17215400 PMC6672079

[B99] Piek J. P. Dyck M. J. Nieman A. Anderson M. Hay D. Smith L. M. (2004). The relationship between motor coordination, executive functioning and attention in school aged children. Arch. Clin. Neuropsychol. 19, 1063–1076 10.1016/j.acn.2003.12.007 15533697

[B100] Phillips J. G. Stelmach G. E. Teasdale N. (1991). What can indices of handwriting quality tell us about Parkinsonian handwriting? Hum. Mov. Sci. 10, 301–314 10.1016/0167-9457(91)90009-m

[B101] Plotnik M. Giladi N. Dagan Y. Hausdorff J. M. (2011). Postural instability and fall risk in Parkinson’s disease: impaired dual tasking, pacing and bilateral coordination of gait during the “ON” medication state. Exp. Brain Res. 210, 529–538 10.1007/s00221-011-2551-0 21279632

[B102] Poletti M. Bonuccelli U. (2013). Acute and chronic cognitive effects of levodopa and dopamine agonists in patients with Parkinson’s disease: a review. Ther. Adv. Psychopharmacol. 3, 101–113 10.1177/2045125312470130 24167681 PMC3805397

[B103] Poletti M. Emre M. Bonuccelli U. (2011). Mild cognitive impairment and cognitive reserve in Parkinson’s disease. Parkinsonism Relat. Disord. 17, 579–586 10.1016/j.parkreldis.2011.03.013 21489852

[B104] Poletti M. Frosini D. Pagni C. Baldacci F. Nicoletti V. Tognoni G. (2012). Mild cognitive impairment and cognitive-motor relationships in newly diagnosed drug-naive patients with Parkinson’s disease. J. Neurol. Neurosurg. Psychiatry 83, 601–606 10.1136/jnnp-2011-301874 22492216

[B105] Pondal M. Del Ser T. Bermejo F. (1996). Anticholinergic therapy and dementia in patients with Parkinson’s disease. J. Neurol. 243, 543–546 10.1007/bf00886877 8836945

[B106] Price A. Filoteo J. V. Maddox W. T. (2009). Rule-based category learning in patients with Parkinson’s disease. Neuropsychologia 47, 1213–1226 10.1016/j.neuropsychologia.2009.01.031 19428385 PMC2681254

[B107] Probst-Cousin S. Druschky A. Neundorfer B. (2003). Disappearance of resting tremor after “stereotaxic” thalamic stroke. Neurology 61, 1013–1014 10.1212/01.wnl.0000086810.14643.fc 14557586

[B135] Rascol O. Sabatini U. Simonetta-Moreau M. Montastruc L. Rascol A. Clanet M. (1991). Square wave jerks in Parkinsonian syndromes. J. Neurol. Neurosurg. Psychiatry 54, 599–602 10.1136/jnnp.54.7.599 1895124 PMC1014429

[B108] Reiner A. (2010). “Organization of corticostriatal projection neuron types,” in Handbook of Basal Ganglia Structure and Function, eds Steiner H. Tseng K. Y. (New York, NY: Academic Press), 323–340

[B109] Reynolds J. N. J. Hyland B. I. Wickens J. R. (2001). A cellular mechanism of reward-related learning. Nature 413, 67–70 10.1038/35092560 11544526

[B110] Schillaci O. Chiaravalloti A. Pierantozzi M. Di Pietro B. Koch G. Bruni C. (2011). Different patterns of nigrostriatal degeneration in tremor type versus the akinetic-rigid and mixed types of Parkinson’s disease at the early stages: molecular imaging with 123I-FP-CIT SPECT. Int. J. Mol. Med. 28, 881–886 10.3892/ijmm.2011.764 21811760

[B111] Schomaker L. R. B. (1991). Simulation and Recognition of Handwriting Movements: A Vertical Approach to Modeling Human Motor Behavior. (Doctoral Dissertation, Nijmegen University).

[B112] Schroll H. Vitay J. Hamker F. H. (2012). Working memory and response selection: a computational account of interactions among cortico-basal ganglio-thalamic loops. Neural Netw. 26, 59–74 10.1016/j.neunet.2011.10.008 22075035

[B113] Seger C. A. (2008). How do the basal ganglia contribute to categorization? Their roles in generalization, response selection and learning via feedback. Neurosci. Biobehav. Rev. 32, 265–278 10.1016/j.neubiorev.2007.07.010 17919725 PMC2376049

[B114] Sheridan M. R. Flowers K. A. (1990). Movement variability and bradykinesia in Parkinson’s disease. Brain 113, 1149–1161 10.1093/brain/113.4.1149 2397387

[B115] Shine J. M. Matar E. Lewis S. J. G. (2013). Differential neural activation patterns in patients with Parkinson’s disease and freezing of gait in response to concurrent cognitive and motor load. PLoS One 8:e52602 10.1371/journal.pone.0052602 23382821 PMC3559645

[B116] Silberstein P. Pogosyan A. Kuhn A. A. Hotton G. Tisch S. Kupsch A. (2005). Cortico-cortical coupling in Parkinson’s disease and its modulation by therapy. Brain 128, 1277–1291 10.1093/brain/awh480 15774503

[B117] Smith Y. Galvan A. Raju D. Wichmann T. (2010). “Anatomical and functional organization of the thalamostriatal systems,” in Handbook of Basal Ganglia Structure and Function, eds Steiner H. Tseng K. Y. (New York, NY: Academic Press), 381–396

[B118] Smith Y. Raju D. V. Pare J. F. Sidibe M. (2004). The thalamostriatal system: a highly specific network of the basal ganglia circuitry. Trends Neurosci. 27, 520–527 10.1016/j.tins.2004.07.004 15331233

[B119] Steiner H. Tseng K. Y. (2010). Handbook of Basal Ganglia Structure and Function. New York: Academic Press

[B120] Tessitore A. Amboni M. Cirillo G. Corbo D. Picillo M. Russo A. (2012). Regional gray matter atrophy in patients with Parkinson disease and freezing of gait. AJNR Am. J. Neuroradiol. 33, 1804–1809 10.3174/ajnr.a3066 22538070 PMC7964749

[B121] Teulings H. L. Stelmach G. E. (1991). Control of stroke size, peak acceleration and stroke duration in Parkinsonian handwriting. Hum. Mov. Sci. 10, 315–334 10.1016/0167-9457(91)90010-u

[B122] Vakil E. Herishanu-Naaman S. (1998). Declarative and procedural learning in Parkinson’s disease patients having tremor or bradykinesia as the predominant symptom. Cortex 34, 611–620 10.1016/s0010-9452(08)70518-5 9800094

[B123] Vandenbossche J. Deroost N. Soetens E. Spildooren J. Vercruysse S. Nieuwboer A. (2011). Freezing of gait in Parkinson disease is associated with impaired conflict resolution. Neurorehabil. Neural Repair 25, 765–773 10.1177/1545968311403493 21478498

[B124] Van Gemmert A. W. A. Adler C. H. Stelmach G. E. (2003). Parkinson’s disease patients undershoot target size in handwriting and similar tasks. J. Neurol. Neurosurg. Psychiatry 74, 1502–1508 10.1136/jnnp.74.11.1502 14617705 PMC1738235

[B125] Van Gemmert A. W. A. Teulings H. L. Contreras–Vidal J. L. Stelmach G. E. (1999). Parkinson’s disease and the control of size and speed in handwriting. Neuropsychologia 37, 685–694 10.1016/s0028-3932(98)00122-5 10390030

[B126] Weinberger M. Hutchison W. D. Lozano A. M. Hodaie M. Dostrovsky J. O. (2009). Increased gamma oscillatory activity in the subthalamic nucleus during tremor in Parkinson’s disease patients. J. Neurophysiol. 101, 789–802 10.1152/jn.90837.2008 19004998

[B127] Weiss P. Stelmach G. E. Hefter H. (1997). Programming of a movement sequence in Parkinson’s disease. Brain 120, 91–102 10.1093/brain/120.1.91 9055800

[B128] Wickens J. R. Begg A. J. Arbuthnott G. W. (1996). Dopamine reverses the depression of rat corticostriatal synapses which normally follows high-frequency stimulation of cortex in vitro. Neuroscience 70, 1–5 10.1016/0306-4522(95)00436-m 8848115

[B129] Wilson C. J. (2004). “Basal ganglia,” in The Synaptic Organization of the Brain, ed Shepherd G. M. (New York: Oxford University Press), 361–414

[B130] Yeterian E. H. Pandya D. N. (1993). Striatal connections of the parietal association cortices in rhesus monkeys. J. Comp. Neurol. 332, 175–197 10.1002/cne.903320204 8331211

[B131] Yeterian E. H. Pandya D. N. (1995). Corticostriatal connections of extrastriate visual areas in rhesus monkeys. J. Comp. Neurol. 352, 436–457 10.1002/cne.903520309 7706560

[B132] Yeterian E. H. Pandya D. N. (1998). Corticostriatal connections of the superior temporal region in rhesus monkeys. J. Comp. Neurol. 399, 384–402 10.1002/(sici)1096-9861(19980928)399:3<384::aid-cne7>3.0.co;2-x 9733085

[B133] Yin H. H. Ostlund S. B. Knowlton B. J. Balleine B. W. (2005). The role of the dorsomedial striatum in instrumental conditioning. Eur. J. Neurosci. 22, 513–523 10.1111/j.1460-9568.2005.04218.x 16045504

[B134] Zaidel A. Arkadir D. Israel Z. Bergman H. (2009). Akineto-rigid vs. tremor syndromes in Parkinsonism. Curr. Opin. Neurol. 22, 387–393 10.1097/wco.0b013e32832d9d67 19494773

